# Distinguishing Dyslexia, Attention Deficit, and Learning Disorders: Insights from AI and Eye Movements

**DOI:** 10.3390/bioengineering12070737

**Published:** 2025-07-05

**Authors:** Alae Eddine El Hmimdi, Zoï Kapoula

**Affiliations:** Orasis-Eye Analytics & Rehabilitation Research Group, Spinoff CNRS, 12 Rue Lacretelle, 75015 Paris, France; alae-eddine.el-hmimdi@etu.u-paris.fr

**Keywords:** saccade, vergence, dyslexia, scholar disorders, attention deficit, machine learning

## Abstract

This study investigates whether eye movement abnormalities can differentiate between distinct clinical annotations of dyslexia, attention deficit, or school learning difficulties in children. Utilizing a selection of saccade and vergence eye movement data from a large clinical dataset recorded across 20 European centers using the REMOBI and AIDEAL technologies, this research study focuses on individuals annotated with only one of the three annotations. The selected dataset includes 355 individuals for saccade tests and 454 for vergence tasks. Eye movement analysis was performed with AIDEAL software. Key parameters, such as amplitude, latency, duration, and velocity, are extracted and processed to remove outliers and standardize values. Machine learning models, including logistic regression, random forest, support vector machines, and neural networks, are trained using a GroupKFold strategy to ensure patient data are present in either the training or test set. Results from the machine learning models revealed that children annotated solely with dyslexia could be successfully identified based on their saccade and vergence eye movements, while identification of the other two categories was less distinct. Statistical evaluation using the Kruskal–Wallis test highlighted significant group mean differences in several saccade parameters, such as a velocity and latency, particularly for dyslexics relative to the other two groups. These findings suggest that specific terminology, such as “dyslexia”, may capture unique eye movement patterns, underscoring the importance of eye movement analysis as a diagnostic tool for understanding the complexity of these conditions. This study emphasizes the potential of eye movement analysis in refining diagnostic precision and capturing the nuanced differences between dyslexia, attention deficits, and general learning difficulties.

## 1. Introduction

Dyslexia, learning disorders, and attention deficits affect a large number of children today. Solid evidence highlights the presence of abnormal eye movement in children or adolescents diagnosed with dyslexia by multidisciplinary medical centers where comprehensive testing is performed, including neuropsychological and pathological assessments.

For instance, Ward and Kapoula recently demonstrated that saccade and vergence eye movements, tested with the REMOBI and AIDEAL technologies, exhibit multiple abnormalities in dyslexic adolescents compared to non-dyslexics. Specifically, people with dyslexia show slower saccadic velocity, poor binocular coordination, and unstable fixations due to disconjugate eye drifting [1]. Vergence eye movements in people with dyslexia also present abnormalities, including smaller amplitudes, lower velocities, and higher variability. Other studies use eye-tracking systems to record, and analyze eye movement in order to characterize eye movement patterns during reading in children [2], or to screen cognitive decline early in patients with Alzheimer’s disease by studying eye movement parameters such as amplitude, duration, and pupil size [3]. Focusing on children with dyslexia, El Hmimdi et al. [4] utilized the same eye movement database as in study [1] and applied linear and non-linear machine learning models to classify dyslexic and non-dyslexic adolescents. Their results showed an accuracy up to 81.25%, with specificity and sensitivity 82.5% and 80.0%, respectively, based on saccade eye movement parameters. Moreover, they predicted reading speed with an error of only 16%. These findings further reinforce the notion that eye movement abnormalities can serve as reliable biomarkers for detecting dyslexia.

Clinicians such as orthoptists, optometrists, and neurologists who work with children and adolescents facing reading problems often describe their conditions using terms like dyslexia, school learning disorder, or attention deficit. A critical question arises: do these terms correspond to distinct types of eye movement abnormalities? In other words, if eye movements are a biomarker of dyslexia, would it be possible to distinguish dyslexia well from other similar conditions?

In another study, El Hmimidi et al. [5] utilized a large clinical dataset from multiple European centers employing the same REMOBI and AIDEDAL technologies, and eye-tracking technology to assess saccades and vergence movements across a large spectrum of patients. The dataset included various populations labeled with terms like dyslexia, learning disorders, attention deficits, school difficulties, and other neurological or psychological disorders, visual fatigue, vertigo, strabismus, etc. In addition to machine learning, deep learning approaches were applied to the time series of eye movements taken end to end. All clinical data related to reading (annotated as dyslexia, reading problems, school learning, and attention deficits) were grouped together. The machine or deep learning algorithms were trained to distinguish such a globally defined group, presented as having school learning disorders, from other groups of pathologies. School learning disorders were successfully classified with either approach, with deep learning performing better but offering lower physiological interpretability. Here, we emphasize the fact that no differentiation was made between subclasses of dyslexia, attention deficit, and learning problems in that study. Research from other groups, although increasingly focused on the integration of eye-tracking technologies and machine learning to advance the assessment and diagnosis of neurodevelopmental and psychiatric disorders, does not address such subtle aspects of annotation uncertainty that are important in the clinic. Next, we will provide a brief review of such research, generally focused on detecting one pathology versus no pathology.

Nilsson Benfatto et al. [6] demonstrated that specific eye movement patterns during reading can effectively differentiate between dyslexic and non-dyslexic readers, supporting the use of eye tracking as a screening tool. Mézière et al. [7] extended the application of eye tracking to reading comprehension. Their study showed that eye-tracking measures can offer valuable insights into comprehension processes, suggesting potential for broader educational assessment.

Svaricek et al. [8] introduced INSIGHT, a two-phase dyslexia detection method that combines fixation visualizations with neural networks. By leveraging eye-tracking data, INSIGHT provides an objective and automated approach that advances beyond traditional diagnostic methods. Vaitheeshwari et al. [9] developed a virtual reality (VR) reading environment that captures detailed eye movement metrics, word vectors, and saliency maps. According to the authors, their fusion model, integrating multiple machine learning algorithms, significantly enhances the accuracy and efficiency of dyslexia diagnosis, marking a notable advancement in educational and assistive technology.

Nerušil benfatto et al. [10] proposed a holistic approach using convolutional neural networks (CNNs) to process entire eye-tracking records in both time and frequency domains. Their method achieved an impressive 96.6% accuracy on a dataset of 185 subjects, demonstrating the power of deep learning in extracting complex patterns from eye movement signals. Jothi Prabha and Bhargavi [11,12] focused on extracting a small set of eye movement features—such as fixations and saccades—using statistical and threshold-based algorithms. Various machine learning models, including hybrid SVMs, random forests, and k-nearest neighbors, were evaluated, consistently showing that well-chosen features can yield robust dyslexia classification.

El Hmimdi et al. [4,13,14] explored the predictive power of eye movement data across diverse tasks, including reading, non-reading, and free painting viewing. Their models achieved accuracies ranging from 70% to over 81%, with features such as velocity, duration, and disconjugacy proving highly predictive. The 2024 study introduced a novel self-learning task (ORASIS-MAE) to improve robustness against eye-tracking failures, by learning to characterize the eye movement response relatively with regard to the stimulus signal, thereby enhancing model reliability.

Importantly, the studies conducted by El Hmimdi et al. are unique in expanding eye movement analysis beyond saccades and fixations to include vergence eye movements, which are essential for reading (Ward& Kapoula [1]). Moreover, El Hmimdi et al. [5,14] compared populations with learning disorders to other clinical populations, rather than limiting comparisons to healthy only; they use multicentric data coming from different clinical centers all using the same technologies (REMOBI and AIDEAL). These studies hold particularly high clinical value as they are more physiologically plausible and represent a significant step forward in connecting eye movement neuroscience with artificial intelligence.

When considering autism and depression, Cilia et al. [15] and Kanhirakadavath & Chandran [16] applied eye tracking and machine learning to ASD screening. Both studies found that characteristic eye gaze and scan path patterns, when analyzed with machine learning, can support early and objective ASD identification. Stolicyn et al. [17] demonstrated that face and eye movement tracking during cognitive tasks can predict elevated symptoms of depression with clinically relevant accuracy (80%), suggesting new avenues for mental health screening. Collectively, these articles illustrate a paradigm shift in the assessment of neurodevelopmental and psychiatric disorders. The integration of eye tracking with advanced machine learning models provides objective, scalable, and non-invasive tools for diagnosis and assessment. Future research should focus on expanding datasets, improving model robustness, and translating these advances into real-world clinical and educational settings.

Returning to the issue of the specificity of dyslexia, the purpose of this study is to test using machine learning as it provides better physiological interpretability, relatively to deep learning algorithms approach, whether clinical annotations emphasizing terms like dyslexia versus attention deficits versus school learning difficulties might reflect different types of dysfunctions or different degree of severity of dysfunction. This study utilizes a selection of the clinical dataset of saccade and vergence eye movements recorded from the 20 clinical centers across Europe, all using the REMOBI and AIDEAL technologies. We selected only data for which clinicians used a single annotation of one of the following three: dyslexia, school learning problems, or attention deficit. It should be noted that the use of multiple annotations, e.g., dyslexia, attention deficit, and school learning disorders, also reflects the fact that all these disorders may coexist in several children; in other cases, the use of multiple annotations could reflect uncertainty about the exact diagnosis. Therefore, in the present study, we aimed to delineate some of these aspects. Mainly, we aimed to respond to the question whether dyslexia annotation alone carries eye movement specificity that distinguishes it even from other related or less well-defined dysfunctions such as learning school issues and attention problems.

The study employs multiple statistical and machine learning classification techniques. Although a statistical approach is valid and widely used in neuroscience, the machine learning approach provides a more holistic and quantitative perspective, as it considers all eye movement features simultaneously. The aim is to compare these methods in a meaningful way to better understand eye movement differences in individuals with dyslexia relative to other populations with school learning problems or attention deficits. Statistical comparisons are performed pairwise to identify which individual eye movement parameters are statistically significant for distinguishing between the different groups.

The results show that children annotated as having dyslexia can only be successfully identified with machine learning applied on their saccade and vergence eye movements, while identification of the other two categories is more loose. Such findings underscore the importance of using eye movement analysis as a diagnostic tool and emphasize the value of specific terminology to capture the complexity of these conditions.

Such findings underscore the importance of using eye movement analysis as a diagnostic tool and emphasize the value of specific terminology to capture the complexity of these conditions.

## 2. Materials and Methods

### 2.1. REMOBI and AIDEAL

REMOBI and AIDEAL technologies enable the testing and analysis of eye movements in both direction and depth, specifically assessing saccades (left/right) as well as convergence and divergence along the median plane. REMOBI is a multisensory visual and acoustic tablet featuring RGB LEDs coupled with a buzzer adjacent to each LED. LEDs and buzzers are aligned along four arcs of isovergence, positioned at distances of 20, 36, 60, and 100 cm from the eyes, allowing for precise stimulation and measurement of binocular motor function (for further details, see (Ward & Kapoula, 2020 [1]). Eye movements are recorded binocularly using the wearable Pupil Core device.

AIDEAL is a cloud-based software platform that enables automatic analysis of various eye movement parameters, including latency, accuracy, amplitude, velocity, and binocular coordination for all types of eye movements. Together, REMOBI and AIDEAL provide robust solutions for functional exploration, diagnosis, and neuro-rehabilitation of binocular vision. REMOBI is protected under patent WO2011073288, and AIDEAL under PCT/EP2021/062224; both are proprietary technologies of Orasis-Ear.

### 2.2. Eye Movement Recording

The different recordings are performed in multiple European clinical centers. The different centers all used the same technologies, REMOBI and AIDEAL, to stimulate and analyze eye movement.

Eye movements are recorded with the Pupil Core device [18], while saccade or vergence tests are performed using the REMOBI technology. The saccade test enables the recording of left and right saccade eye movements, while the vergence test enables the recording of convergent and divergent eye movements.

During the recording, the stimulus target signal is stored along with the instantaneous eye movement signal to allow further analysis of the eye movement relative to each stimulus.

During the saccade test, the target light emitting diode (LED) was located eccentrically on the horizontal axis at 16 degrees to the left or to the right in a pseudorandom way for 1.5 to 2 s. Then, the center LED was turned on to guide the eye back to the center. Similarly, during the vergence test, the convergence LED was presented at the center at a viewing distance of 20 cm from the person’s eyes, while the divergence LED was presented at 100 cm, also centrally; then, the target LED was switched off and the initial fixation LED located centrally at 36 cm was turned on again.

Notably, almost the complete range of eye movement parameters is used in the analysis. These include amplitude, which describes the accuracy of the movement relative to the target; latency, which indicates the time required by the brain to program the motor command; duration; and peak velocity and average velocity, which assess the dynamics of movement execution. Additional parameters, known to be impaired in dyslexia, include the quality of binocular coordination, evaluated as disconjugacy (the difference in amplitude between the two eyes during a saccade), as well as disconjugate drifts of the eyes during the first 80 ms after the end of a saccade (drift 1), and over the total 160 ms following saccade offset. All these parameters are essential, as increased disconjugacy and drifts are detrimental to clear, fused vision (Ward & Kapoula [1]).

Importantly, these parameters are extracted automatically by the AIDEAL software V3, making the analysis independent of the operator. The operator (from various clinical centers) records the eye movements and sends the data file, which contains the eye position and LED information, to AIDEAL for analysis.

#### Ora22 Dataset

We used a selection of data extracted from the larger dataset Orasis 2022 (Ora22) previously used by [5]. We selected 355 individuals in the saccade tests with one of the three annotations cited before, and 454 individuals in the vergence task. The count of each of the three classes is presented in Table 1, along with their corresponding age mean and the median, for each of the saccade and the vergence visual tasks. The dyslexia group is larger, followed by scholar disorder annotation, and finally the attention deficit annotation.

**Figure A1:**
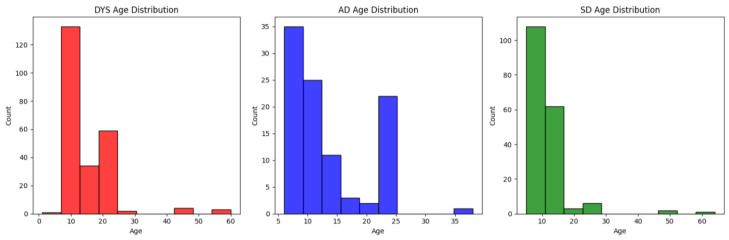
Presentation of the age histogram of the population, for each of dyslexic (Dys), attention deficit (AD), and scholar disorder (SD) group.

We present in Figure A1 of the Appendix B the distribution of each of the three classes, namely dyslexia (DYS), scholar disorder (SD), and attention deficit (AD).

Overall, the populations for the two eye movement tasks present similar median and standard deviation statistics, except for the SD population, which has approximately twice the standard deviation. The three populations have a median value between 10 and 12; however, the dyslexia population shows the highest variability, while SD has lower variability in the saccade task.

Thus, the three groups correspond to a population with a comparable age, all of them under the age of 30, with a few exceptions. Furthermore, the age distribution for AD is bimodal, while the two other groups (SD and DYS) consist mainly of children.

Furthermore, we present in Table 1 the count of each of the three populations, along with their corresponding mean and median age, for both the saccade and vergence visual tasks separately.

### 2.3. Eye Movement Analysis

Each recording is analyzed with AIDEAL software analysis, processing each eye movement time series relative to the stimulus target signal. For the saccade task, the horizontal conjugate signal was computed using the mean of the horizontal left and right eye position time series to analyze each recording. On the other hand, for the vergence task, we use the disconjugate signal, i.e., the left minus right eye position time series difference. First, using the stimulus signal, the software extracts (for each trial) the onset and the offset of the target signal, as well as the onset and the offset of the eye movement signal for the saccade and for the vergence test. The latency of the eye movement and its amplitude relative to the LED target onset is evaluated; movements that are of a very low amplitude (low than 30% of the requirement) or are abnormal (too fast or too slow latency) are automatically rejected by the AIDEAL software. These are standard criteria used in the eye movement neuroscience field.

For the retained eye movements, the different eye movement parameters are computed for each trial and then aggregated using the mean and standard deviation operators, resulting in 32 parameters for the saccade task and 36 parameters for the vergence task. We present in Table A2 of the Appendix A the different eye movement parameters used for each of the tests.

### 2.4. Data Processing

In addition to the processing performed by the software analysis, two additional processing steps are introduced to address outliers and standardize the different parameter values.

First, to remove outliers, we substitute all values with a Z-score below 2.5. In other words, for each parameter, we compute its corresponding mean *m* and standard deviation std; then, we have

ifx<m−2.5×std,x←m−2.5×std

ifx>m+2.5×std,x←m+2.5×std

Then, we standardize each parameter by substituting each value *x* with its corresponding mean parameter and dividing by the standard deviation of the corresponding parameter:

$$x_{\mathrm{standardized}}=\frac{x-m}{\mathrm{std}}$$

Moreover, note that each of the two processing steps is performed differently during each training cross-fold iteration, using only the training data of the corresponding fold [19].

### 2.5. Model Training

To evaluate our model, we aimed to utilize a split strategy that takes into account the identity of the recording to ensure that each patient’s data are present either in the training or the test set data. For instance, each subject has one or two recordings. Thus, using classic cross-validation [20] may lead to splits where the same subject appears in both the train and test sets. To address this, we use GroupKFold, which ensures that each group’s data are either entirely in the training folds or entirely in the test folds. In our study, the group attribute is set to the patient identifier.

We fit five models: logistic regression (LR) [21], random forest (RF) [22], support vector machine with an RBF kernel (SVM-RBF) [23], and two neural network variants (MLP and MLP-tanh) [24]. We use the scikit-learn implementation with default parameters.

Moreover, to mitigate the impact of high class imbalance on the training dynamics, we use the balanced feature, which allows the model to reweigh the per-observation loss according to class frequency.

Finally, for the neural network, we use a three-layer architecture with 64, 64, and 32 neurons. We use the Adam optimizer with adaptive learning, training for a maximum of 500 iterations. The only difference between the two variants is the activation function: in the first model, we use ReLU activation; in the second model, we use Tanh activation. Similarly, we use the scikit-learn implementation.

### 2.6. Threshold Tuning

Initially, we observe that the model’s performance suffers due to the high class imbalance, as the default 50% threshold used to convert classification probabilities into decisions is biased. Therefore, we perform a second step at the end of each training fold, aiming to optimize balanced accuracy in order to equalize the performance across both classes.

We follow the approach used in [25] by using the Tuned Threshold ClassifierCV [26] implementation provided in the scikit-learn library.

### 2.7. Model Evaluation

To evaluate the performance of our model, we consider three metrics: the F1 score, specificity, and the sensitivity. During each cross-validation iteration, the model is evaluated on the remaining evaluation fold, and the different scores are aggregated to form the final evaluation score. Finally, the different model performances are ranked using sensitivity and then specificity, if each of these two metrics is greater than 0.5; otherwise, the macro F1 score is considered.

Moreover, we found that the performance varies; thus, for each metric, we report the performance of each class separately.

## 3. Results

### 3.1. Statistical Evaluation of Group Mean Differences

#### Saccades

Table 2 and Table 3 present, respectively, the mean group values for each saccade parameter, for saccades to the left, saccades to the right, and for vergence (divergence and convergence);a comparison of means between different groups was carried out with the Kruskal–Wallis test, and statistically significant differences are indicated by bold values.

The comparison of saccade parameters indicates multiple statistically significant differences, mainly in the comparison between subjects with dyslexia and those with school learning disorders or attention deficits. Specifically, only 4 out of the 34 parameters of saccades and vergence shown in Table 2 and Table 3 are statistically significant when comparing subjects with an attention deficit and dyslexia. Similarly, when comparing subjects with attention deficit and school learning disorders, only 4 out of 34 parameters show significant differences. In contrast, when comparing subjects with dyslexia with those with school learning disorders, 16 out of the 34 parameters differ significantly.

Importantly, saccade latencies to the left and right, as well as divergence latency, are significantly longer in subjects with dyslexia. Additionally, the peak velocity of saccades to the left, to the right, and during convergence is lower in people with dyslexia. Moreover, the average velocity of divergence is lower in people with dyslexia, and the amplitude of both convergence and divergence is also reduced compared to subjects with school learning disorders. These observations align with previous studies comparing individuals with dyslexia with healthy controls, indicating slower latencies and velocities as well as vergence limitations. Interestingly, similar parameters differentiate dyslexic children from those with school learning disorders.

The combined results of prior and current studies suggest the existence of specific eye movement abnormalities in individuals with dyslexia. In the next step, the classification and differentiation of various groups (dyslexia, attention deficit, or school learning disorders) will be performed using machine learning models. The advantage of this approach is that it considers all eye movement parameters together rather than analyzing them separately.

### 3.2. Machine Learning Approach

Below, we present the different model performance when training machine learning models to screen the three classes. Recall that, in this study, we explore screening each of the following three classes, dyslexic, attention deficit, and scholar disorder, which is more challenging relative to screening each of these classes among healthy control populations. Moreover, the using data from multiple clinical centers presents variability, making the learning task more challenging. However, these situations correspond to the real model inference condition, as the model is intended to be deployed in multiple clinical centers, and should be able to screen these populations, not only among healthy populations but also among other populations with similar pathologies.

#### 3.2.1. Saccade Visual Task

We present the different metric scores in Table 4 when learning to screen each of the three pathologies using the saccade dataset.

When considering the overall performance, SVM achieved the best average F1 score of 48.7%. On the other hand, when considering the per-class order of performance, the different models achieved varying performance in screening dyslexia, relative to screening SD and screening AD. The highest performance was achieved in screening dyslexia with an F1 score of 61.54%, while the lowest performance was achieved when screening AD using the tanh-based neural network, with an F1 score of 30.76%.

Finally, when considering the models’ performance in terms of trade-offs between the ability to screening positive and negative classes, despite using threshold tuning, only the random forest method showed balanced performance, but at the cost of a lower F1 score.

#### 3.2.2. Vergence Visual Task

Similarly, we present the different metric scores in Table 5 when learning to screen each of the three pathologies using the vergence dataset.

Overall, the order of performance for vergence is similar to the performance for saccades, with the SVM achieving the best F1 score for screening dyslexia. On the other hand, the neural network shows the lowest performance when screening AD.

In contrast, the neural network performs better in screening relative to both the random forest and logistic regression. It achieves an F1 score of 59.0% for screening AD. Additionally, this model shows the best sensitivity and specificity trade-off (58.1% and 51.3%, respectively).

## 4. Discussion

Eye movements are a sensitive biomarker reflecting reading capacity, which is essential for learning in school. The purpose of this study was to explore whether eye movement properties can enable the subtle classification of children clinically annotated as dyslexic, those with more general annotations concerning school learning disorders, and those diagnosed with an attention deficit.

The dataset for this study was carefully selected from a larger pool to ensure no overlap between categories. It was critical that clinicians assigned precise annotations to the children, choosing the most appropriate labels among various possibilities. Therefore, we believe these datasets represent distinct clinical identities.

The central question is whether eye movement properties differ among these subpopulations and if they enable classification into the three subgroups using machine learning (ML) approaches. Before applying ML algorithms, classic statistical analysis of all eye movement parameters demonstrated significant differences, especially between the dyslexic group and the school learning disorders’ group, with fewer differences observed in the other pairwise comparisons.

For instance, several parameters related to saccades showed significant differences between the dyslexic and school learning disorder groups, more so than in any other comparison. This was consistent for both saccade and vergence eye movements. Notably, the differences between dyslexic and school learning disorder groups were related to latency and velocity profiles.

The previous study by Ward and Kapoula [1] included 45 children with dyslexia compared to 42 healthy children, while the current study expanded the dataset to 247 children, with all of them presenting some clinical concerns; also, data were collected from various clinical centers across Europe. In the previous study, data were collected at two schools by the same researchers. This broader dataset confirms that the velocity and latency of eye movements in people with dyslexia exhibit a specific pattern, differentiating them even from other clinical populations; note that velocity and latency reflect functioning of the brainstem and cerebral circuits, respectively, responsible for the execution and programming of saccade and vergence eye movements.

The addition of machine learning analyses provided further insights, as it considers all parameters together and utilizes both linear and non-linear models. The school learning disorder group, although distinct, was also relatively well classified. In contrast, the attention deficit group showed ambiguous results, indicating that it does not form a clear, distinct class.

Overall, the findings are significant as they confirm specific eye movement problems in dyslexia, distinguishing it from general school learning disorders. While the neurological basis of dyslexia remains debated, this study supports the hypothesis of distinct functional characteristics in the eye movement generators at both the brainstem and cortical levels in dyslexia.

### 4.1. Limitations

While the approach taken here is used to identify specific eye movement patterns associated with precise diagnoses and single annotation, it also presents a limitation in reflecting the clinical reality where these disorders frequently coexist, or where the diagnosis might be uncertain and multiple annotations are used. The decision to exclude individuals with comorbid conditions, although being a method used to ensure distinct clinical identities, might restrict the generalizability of the findings to the broader population of children with learning difficulties.

Furthermore, the sample sizes differ across the three groups and between the saccade and vergence tasks, with dyslexia generally having the largest group. The age distribution is also noted to vary, with the attention deficit group showing a bimodal distribution, unlike the other two which mainly consist of children. Further studies with larger groups are of interest. Finally, in the studies, we use the default hyperparameters of the scikit-learn library. Optimizing these hyperparameters can improve the different parameters; however, one should optimize them using a separate validation set to avoid introducing bias into the validation results. On the other hand, with a small dataset, splitting subjects into train, test, and validation sets is challenging. Thus, increasing the dataset size to tackle these different limitations is a future direction.

### 4.2. Machine Learning or Deep Learning?

When considering analyzing eye movement using AI algorithms, should we either opt for features defined using machine learning or allow the algorithm to define its own relevant set of parameters? In other words, machine learning uses a few critical points of eye movement signals based on velocity criteria, establishing the onset and the offset of each movement and the fixation periods between the movements.

In contrast, deep learning processes signals end-to-end over time; features are not imposed based on expertise and the algorithms themselves have to find characteristics of the signals, enabling us to discriminate between different populations. When considering eye movement analysis, several studies explore automatizing screening analysis using machine learning algorithms [7,17] and deep learning algorithms [5,14,15], or compare the two approaches [5,16]. When considering the machine learning approach, previous studies have explored screening dyslexia using eye movement parameters computed during reading and stimulus-driven tasks [13] and during context-free exploration tasks [4]. Screening dyslexia using machine learning has been more extensively explored in the literature, including [6,12].

When considering deep learning, some studies on eye movements exist mostly on dyslexia [10,27] and autism spectrum disorder. Overall, ML studies have been carried out more frequently than DL; thus, future research should include DL. It should be noted that studies comparing ML and DL performance using research and clinical data [16] to screen for autism on the basis of eye scan paths over images found that DL methods achieved better scores. Similarly, in our previous studies [5], we found better performance when experimenting with screening learning disorders from time series data with a DL approach.

While deep learning methods demonstrate promising performance, abstract feature extraction with the DL approach remains a major limitation. In the medical domain, using scientific expertise-based parameters for screening is more accepted than robust but less interpretable solutions. Still, future research could further investigate these specificities using end-to-end deep learning approaches combined with visualizations of pattern detection. Such methods that are ongoing in our laboratory can capture subtle differences between different clinical populations and provide new insights.

Other major issue concerns comprise the clinical utility of AI approaches in handling populations with infra clinic eye movement abnormalities, populations with uncertain annotations, and so on. Future developments in AI-assisted tools could help clinicians refine their annotations and contrast DL- and ML-based classifiers. We believe that AI applied to eye movements can be a powerful tool used to advance both our scientific understanding and clinical practice.

### 4.3. Perspective AI and Neuroscience

Despite limitations, the present study presents promising responses for both these issues: physiological interpretability of AI data and usefulness of future AI assistance in clinical practice. It is important to highlight that in our studies, we find the same eye movement parameters differentiating people with dyslexia from healthy individuals without it or even from other pathologies (current study), namely related to the velocity profile of saccades—lower average velocity than in healthy individuals and, as shown here, lower velocities than persons with learning disorders, as well as lower divergence and convergence amplitudes than individuals with learning disorders.

All these aspects are highly dependent on cerebellar fine control, and it has been suggested that mild cerebellar inefficiency or dysfunction might be a hallmark of dyslexia, affecting several other aspects including posture control, equilibrium, language processing, and phonologic processing in a more general way (see Li H et al. [28]).

Recent research continues to support and refine the cerebellar theory of dyslexia, while also integrating it into broader network models of reading and learning disabilities.

Advanced imaging studies confirm that adults with dyslexia exhibit disrupted functional and effective connectivity in the cerebellum, especially the right cerebellum, during reading tasks. These disruptions are linked to poorer reading performance, suggesting that cerebellar dysfunction is a neural marker of dyslexia (Turker [29]).

While some studies (e.g., on children with both reading and math disabilities) did not find significant differences in cerebellar activation, the cerebellar deficit hypothesis has evolved. The delayed neural commitment hypothesis now emphasizes the cerebellum’s role in automatization, timing, and its connections with cortical language areas, rather than isolated cerebellar dysfunction (López-Escribano [30]).

Intervention studies show that combined cognitive and motor training can improve reading and writing abilities in children with dyslexia, likely by enhancing cerebellar function and neural plasticity. These findings suggest practical applications for therapies targeting cerebellar–cortical networks (Fiorilli, C. et al. [31]).

In summary, the cerebellum remains a central focus in dyslexia research, with recent studies clarifying its role within larger brain networks and highlighting the promise of targeted interventions.

In the present study, we also observe longer latency in people with dyslexia. Latency reflects the function of visual parietal–frontal networks that are activated to plan, program the eye movement, and trigger it at the appropriate time to a greater extent; latency thus reflects a series of processes occurring in parallel (see Findlay & Walker [32]).

Thus, parallel execution of such functions and subtending neural networks could be less efficient in people with dyslexia as their latencies were longer for both saccades and vergence relative to subjects with school learning disorders.

AI-driven feature-level classification offers valuable neurological insights. For example, when both classic statistics and machine learning highlight slowed saccade velocity, they support the cerebellar theory of eye movement control. Conversely, when prolonged saccadic latency is detected, they reinforce the hypothesis of inefficiency in the visual–parietal–frontal circuitry responsible for eye movement programming. In this way, AI-based feature analysis emerges as a powerful tool for deepening our understanding of brain function.

Going a step further, now using machine learning and interpretable feature approaches, one could search and run machine learning with specific features, also using deep learning interpretable models, providing typical patterns of velocity and latency profiles, all integrated in an AI assistant. The clinician could thus search for such specific hallmarks to assess and monitor the progress of the patient.

This is a major advantage AI could bring to the clinic. Moreover, new retraining methods could be invented to recalibrate and train those specific aspects, as eye movements are a model of neural plasticity and, with appropriate training, could be largely improved. This opens new avenues for the treatment of these problems, leading to better academic performance. Our ongoing research and developments are focused on these areas.

## 5. Conclusions

In conclusion, this study, which used the ML approach applied on saccade/vergence eye movement tasks collected from many clinical centers all using the same testing acquisition and analysis technology (REMOBI and AIDEAL and Pupil Core), enables us to demonstrate that it is possible to differentiate children with dyslexia from children with school learning issues or attention deficits. This suggests that dyslexia is a distinct case, despite being frequently confounded with annotations such as school learning disorders and attention deficits. Although these problems may coexist, the eye movement signature of dyslexia remains distinct.

## 6. Patents

Zoï Kapoula has applied for patents for the technology used to conduct this experiment.

- **REMOBI** is an embedded device with many paradigms and is used to stimulate and record saccade, vergence, and combined eye movements in 3D space (patent US8851669, WO2011073288)
- **REMOBI Neuro Cog**, EP 4 215 172 A1.
- **AIDEAL** is an eye movement analysis program, WO EP US WO2021228724A;
- A pending patent application concerns **learning disorders**, EP21798945.8A OWO 2021/228724

## Figures and Tables

**Table 1:** Median and standard deviation of age for each of the populations for the saccade and vergence tests.

	Saccade			Vergence		
	**Median**	**Std**	**Count**	**Median**	**Std**	**Count**
Dyslexia	12.0	8.36	189	12.0	8.04	223
Scholar Disorder	10.0	3.19	118	10.0	6.45	174
Attention Deficit	12.0	6.44	77	12.0	6.38	92

**Table 2:** Mean values for each of the three groups are presented, along with the Kruskal–Wallis statistical test used to assess the significance of the differences in the saccade eye movement test. Note that *, **, and *** represent *p*-values below 0.05, 0.001, and 0.0001, respectively.

Parameter		AD vs. DYS			AD vs. SD			DYS vs. SD		
		AD	DYS	Stat	AD	SD	Stat	DYS	SD	Stat
Saccades Left	Amplitude	12.33	11.34	9.02 **	12.16	11.76	0.96	12.16	12.42	2.05
	Latency	230.04	232.37	0	246.08	224.7	4.04 *	245.81	226.91	5.15 *
	Duration	62.7	61.01	1.28	58.62	61.11	1.21	58.49	62.59	9.75 **
	Max. Vel.	332.54	306.84	1.42	271.2	326.05	6.07 *	263.88	335.09	14.7 ***
	Avg. Vel.	176.98	160.94	1.87	177.91	167.1	1.42	178.84	178.53	0.34
	Drift 1	0.66	0.53	0.04	0.71	0.5	2.49	0.74	0.65	2.51
	Drift 2	0.84	1.17	0.16	0.83	1.02	5.74 *	0.86	0.82	6.07 *
	Sacc. Disc.	2.85	2.29	2	2.25	2.32	0.6	2.29	2.85	6.22 *
Saccades Right	Amplitude	12.12	11.91	2.49	12.16	11.86	1.84	12.27	12.14	0.02
	Latency	208.56	217.9	0.65	231.29	218.25	1.83	233.47	210.43	10.3 **
	Duration	61.92	62.29	0.68	58.77	60.81	2.42	57.75	60.91	3.39
	Max Vel.	331.24	294.82	1.76	298.18	304.84	1.1	294.17	330.47	5.44 *
	Avg. Vel.	185.77	163.39	4.32 *	188.84	172.88	2.7	190.94	188.7	0.46
	Drift 1	0.8	0.77	0.1	0.46	0.68	2.08	0.47	0.77	7.12 *
	Drift 2	1.02	1.02	0.28	0.63	0.9	1.49	0.64	0.99	7.23 *
	Sacc. Disc.	2.77	2.45	0.23	2.58	2.44	1.47	2.6	2.76	0.46

**Table 3:** Mean values for each parameter of vergence eye movements for each of the three groups, along with the Kruskal–Wallis statistical test used to assess the significance of the differences between the groups taken two by two. Note that *, **, and *** represent *p*-values below 0.05, 0.01, and 0.001, respectively.

Parameter		AD vs. DYS			AD vs. SD			DYS vs. SD		
		AD	DYS	Stat	AD	SD	Stat	DYS	SD	Stat
Divergence	Phasic amplitude	2.73	2.53	0.35	2.43	2.48	0.29	2.44	2.71	2.6
	Latency	339.12	324.38	2.04	359.34	331.88	7.97 **	360.94	341.54	6.21 *
	Duration	66.37	66.14	0.01	70.25	68.88	0.01	68.83	66.41	0.51
	Max. Vel.	103.12	90.37	0.08	96.96	88.53	0.05	95.09	100.42	0.12
	Avg. Vel.	38.91	37.9	2.16	40.91	34.98	2.77	42.94	39.04	0.11
	Amplitude (80 ms)	0.66	0.67	0.06	0.66	0.59	1.93	0.68	0.65	0.59
	Amplitude (160 ms)	1.18	1.08	1.86	1.11	0.96	2.52	1.16	1.16	0.11
	Ampl. phasic + 160	3.81	3.43	4.62 *	3.36	3.32	0	3.4	3.77	7.77 **
	Avg. Velocity phasic	17.24	15.36	5.67 *	14.77	14.77	0.25	15.02	17.09	13.31 ***
Convergence	Phasic amplitude	3.26	3.35	0	2.79	3.02	0.75	2.86	3.2	7.97 **
	Latency	347.66	340.06	0.79	354.19	348.47	0.48	350.22	347.58	0.07
	Duration	73.62	72.42	0.04	64.49	69.85	3.17	63.14	71.9	10.65 **
	Max. Vel.	130.77	118	0.04	106.24	105.35	0.86	109.55	127.66	5.94 *
	Avg. Vel.	44.34	44.17	0.51	53.23	40.47	1.79	56.15	44.71	0.63
	Amplitude (80 ms)	0.81	0.73	0.27	0.99	0.67	0	1.02	0.79	0.88
	Amplitude (160 ms)	1.47	1.26	0.24	1.51	1.17	0.04	1.55	1.44	2.01
	Ampl. phasic + 160	4.59	4.45	0.07	4.29	4.11	0.46	4.38	4.52	6.2 *
	Avg. Vel. phasic	19.89	19.49	0	19.55	18.14	0.42	20.03	19.68	2.62

**Table 4:** Performance of screening each of the three groups on various metrics for the saccade test in terms of the average (macro) of each of the F1, sensitivity (sens.), and specificity (spec.) scores. The best metric for each class is highlighted in bold.

Model	Metric	DYS	SD	AD	Average (Macro)
Logistic Regression	F1	51.75%	38.5%	34.75%	41.7%
	Sens.	46.15%	45.8%	**59.09%**	50.3%
	Spec.	60.36%	58.2%	48.31%	55.6%
SVM (RBF)	F1	**61.54%**	**49.5%**	35.06%	48.7%
	Sens.	**67.40%**	**75.4%**	58.18%	67.0%
	Spec.	36.49%	40.9%	50.38%	42.6%
Random Forest	F1	52.9%	47.8%	**37.41%**	46.0%
	Sens.	48.0%	61.3%	50.0%	53.1%
	Spec.	59.00%	56.8%	66.49%	60.8%
MLP	F1	43.05%	41.0%	31.54%	38.5%
	Sens.	34.06%	46.5%	48.18%	42.9%
	Spec.	**70.27%**	63.5%	55.06%	62.9%
MLP-tanh	F1	56.2%	39.2%	30.76%	42.0%
	Sens.	57.14%	39.4%	34.54%	43.7%
	Spec.	43.24%	**72.1%**	**74.28%**	63.2%

**Table 5:** Performance of screening each of the three groups on various metrics for the vergence test in terms of the average (macro) of each of the F1, accuracy, sensitivity (sens.), and specificity (spec.) scores. The best metric for each class is highlighted in bold.

Model	Metric	DYS	SD	AD	Average (Macro)
Logistic Regression	F1	42.48%	**48.3%**	**32.2%**	41.0%
	Sens.	35.21%	**67.7%**	**73.2%**	58.7%
	Spec.	65.54%	41.0%	30.7%	45.7%
SVM (RBF)	F1	**64.3%**	46.5%	22.2%	44.3%
	Sens.	**75.41%**	52.3%	18.7%	48.8%
	Spec.	33.33%	61.9%	**87.8%**	61.0%
Random Forest	F1	41.46%	43.8%	28.6%	37.9%
	Sens.	31.89%	47.1%	43.7%	40.9%
	Spec.	**75.28%**	64.3%	60.3%	66.6%
MLP	F1	59.00%	45.5%	20.7%	41.7%
	Sens.	64.78%	49.7%	20.5%	45.0%
	Spec.	38.20%	64.1%	80.9%	61.1%
MLP-tanh	F1	57.75%	42.2%	24.0%	41.3%
	Sens.	58.1%	43.1%	26.8%	42.7%
	Spec.	51.3%	**68.1%**	76.3%	65.2%

## Data Availability

The datasets generated during and/or analyzed during the current study are not publicly available due to privacy restrictions. However, upon reasonable request, they can be made available by the corresponding author.

## Appendix A

**Table A1:** Definitions of the eight acronyms used for different types of eye movement parameters.

Acronym	Definition
RM (CM)	Right mean (Convergent mean)
LM (DM)	Left mean (Divergent mean)
RS (CS)	Right std (Convergent std)
LS (DS)	Left std (Divergent std)

**Table A2:** Presentation of the set of eye movement parameters computed by the software AIDEAL for each trial, depending on the type of test.

Saccade Analysis	Vergence Analysis
Amplitude	Phasic amplitude
Latency	Latency
Duration	Duration
Max. Velocity	Max Vel.
Avg. Velocity	Avg. Vel.
Drift 1	Amplitude (80 ms)
Drift 2	Amplitude (160 ms)
Saccadic Disconjugacy	Amplitude phasic
	Average Velocity phasic + 160 ms

## Appendix C

**Table A3:** Illustration of the joint count, showing the count of occurrences for each pathology pair. Note that the diagonal cells correspond to the count of recordings with only a single annotation.

	Saccade			Vergence		
	AD	DYS	SD	AD	DYS	SD
AD	77	20	4	92	25	5
Dys	20	190	6	25	225	7
SD	4	6	118	5	7	174
